# Effect of Premedication on the Success of Inferior Alveolar Nerve Block in Patients with Irreversible Pulpitis: A Systematic Review of the Literature

**DOI:** 10.1155/2019/6587429

**Published:** 2019-02-10

**Authors:** Meric Karapinar-Kazandag, Jale Tanalp, Handan Ersev

**Affiliations:** ^1^Yeditepe University Faculty of Dentistry, Department of Endodontics, Istanbul, Turkey; ^2^Istanbul University Faculty of Dentistry, Department of Endodontics, Istanbul, Turkey

## Abstract

**Background:**

Failure in the provision of inferior alveolar nerve block anesthesia (IANB) is a significant problem during endodontic treatment of irreversible pulpitis. Various methodologies have been advocated one of which is administration of premedication prior to anesthesia. Despite the considerable number of reports, the topic yet deserves more clarification. This systematic review was conducted to provide an oversight on the effectiveness of premedication prior to IANB in mandibular teeth.

**Methods:**

A PubMed and Cochrane Database search was conducted by using MeSH terms inferior alveolar nerve block + pulpitis and mandibular anesthesia+pulpitis. Two reviewers independently performed the screening, selection of papers, and data extraction. Papers in English language that included randomized clinical studies on the impact of different medications on the success of inferior alveolar block anesthesia in irreversible pulpitis were included. Additionally, relevant supporting literature was also used where necessary.

**Results:**

Initially, 118 papers were selected from PubMed and 68 were selected from Cochrane. Five additional articles were retrieved from Google Search. Following the elimination of duplicates and irrelevant articles, 35 studies were selected meeting the criteria. It was observed that there was moderate evidence to suggest that some premedications were partially effective for the enhancement of mandibular anesthetic effect in irreversible pulpitis.

**Conclusion:**

Though some medications appear to be promising, further supporting research will help highlight this significant topic which requires further clarification.

## 1. Introduction

One of the primary challenges faced by the clinician during endodontic therapy of mandibular teeth is the accomplishment of a successful anesthesia in patients with irreversible pulpitis using the inferior alveolar nerve block (IANB). This poses major difficulty from a clinical point of view as an inadequately anesthetized hot tooth with severe pain will not only lead to elevation of apprehension by the patient but also cause distress in the practicing clinician. Studies reported that the failure ratio of a single IANB block injection of local anesthetic in patients with irreversible pulpitis ranges between 30 and 90 percent [1–3].

Many theories have been proposed as the causative factors for the lack of achievement of successful anesthesia in mandibular teeth with irreversible pulpitis. The hyperalgesia triggered by the inflammatory process leading to alteration of neural response [4], raised levels of prostaglandins and activation of nociceptors [1], lowered pH which hampers the ability of the anesthetic to penetrate the membrane [1, 5], tetrodotoxin resistant sodium channels shown in human symptomatic dental pulp and trigeminal ganglion [6], the sprouting of the nerve fibers [7], and increase in neuropeptides such as Substance P and calcitonin gene-related peptide (CGRP) resulting in the expression of inflammatory mediators [8] are some suggestions made to explain the reason of anesthetic failure associated with acute symptomatic teeth. Nonmyelinated C-fibers which pose difficulty in terms of provision of anesthesia have also been proposed as a contributing factor [9]. Furthermore, anatomical factors such as the mylohyoid nerve have been held responsible for the failure in achievement of a successful anesthesia. It has been suggested that the location of separation of the mylohyoid nerve is away from the injection site and inferior alveolar block anesthesia may not be sufficient enough to be effective on these fibers [10]. Finally, it has also been reported that apprehensive patients with lower pain thresholds are more likely to cause difficulty in obtaining a sufficient anesthetic effect [11].

The issue of difficulty in the achievement of anesthesia in molar teeth has been addressed frequently by researchers and solutions have been proposed such as the utilization of alternative anesthetics [12, 13], supplemental injections [14], alteration of epinephrine ratio [15], alteration of volume of anesthetics [16, 17], and utilization of different compounds and additives in anesthetics and addition of medications in anesthetics or administration of medications preoperatively [18–20].

Confronted with these challenges, many studies have been performed on this issue which has not yet been adequately clarified. Pain perception takes its origin from peripheral neurons that are also known as nociceptors. One of the mechanisms that may cause increased pain perception might be the alterations in the electrical excitability of neurons. Voltage gated ion channels play a crucial role in the determination of the excitability of neurons. Many studies were performed in the last 2 decades that evaluate the impact of channels on the nociception, hyperexcitability, and increased pain sensitivity [21, 22]. Changes in the activities of many different ion channel types were shown in different pain models and there is evidence to support that a few types of ions may play a significant role in nociception and pain sensitivity. Nine different types of voltage-gated sodium channels have been isolated from mammalians [23]. The majority of the currents of tetrodotoxin-resistant (TTX-R) sodium channels are transmitted via Nav 1.8 channels. The Nav 1.8 currents are activated slower and inactivated faster compared to tetrodotoxin-sensitive (TTX-S) currents [24]

A major focus of attention has been put on the reduction of inflammation prior to local anesthesia to enhance the success of anesthetics. Inflammation has been regarded as one of the important factors that play a role in failed anesthetics as mediators of inflammation have the potential to stimulate nociceptor fibers even at very low thresholds and it has been stated that decreasing the amount of prostaglandins may increase the efficacy of local anesthetics [25].

Nav 1.9 contributes to the hypersensitivity caused by inflammatory mediators on the peripheral endings of nociceptors. Consequently, it plays a major role in peripheral sensitization [26].

Consequently, attempts have been made to seek the best medication or combination of medications to be administered prior to endodontic procedures to alleviate inflammation and decrease mediators which are the major causes of painful symptoms. However, although some medications are promising, there appears to be no consensus between authors regarding this clinically important issue.

This article focuses on the studies performed on this topic and aims to provide an overview of the current knowledge we have regarding premedication in irreversible pulpitis to facilitate the anesthetic effect. Randomized controlled clinical trials evaluating the success rate of IANB in patients with irreversible pulpitis in mandibular posterior teeth and administered with various medications and placebo were searched and results of different studies were compared with each other. Clinical studies including premedication with NSAIDs (ibuprofen and others), acetaminophen, corticosteroids, opioid analgesics, benzodiazepines, nitrous oxide and other sedatives, hyperosmolar solutions, and antihypertensive medications and magnesium sulfate were included.

## 2. Materials and Methods

This systematic review followed the PRISMA guidelines [27].

The focused question was given as follows: Is premedication performed prior to endodontic treatment effective in enhancing the anesthetic efficacy of inferior alveolar nerve block in patients with irreversible pulpitis?

Therefore, the PICO was as follows.

Patients (P): they were adult individuals over 18 years old.

Intervention (I): it was medications or placebo administered to patients prior to the administration of inferior alveolar nerve block anesthesia

Comparison (C): there were different types of medications classified under the groups such as NSAIDs, acetaminophen, corticosteroids, opioid analgesics, benzodiazepines, different groups of narcotic analgesics, nitrous oxide and other sedatives, hyperosmolar solutions, and antihypertensives and magnesium sulfate.

Outcome (O): it was any favorable and significant increase in the anesthetic efficacy due to the administration of premedications by VAS scores reported by patients.

### 2.1. Information Sources and Search Strategy

We developed appropriate search strategies for each database we searched.

Literature searches were performed using the Cochrane Library database and PubMed. The following keywords were used: ‘inferior alveolar nerve block' and ‘irreversible pulpitis' in searching Cochrane Central Register of Controlled Trials: Issue 2 of 12, February 2018 and PubMed (irreversible[All Fields] AND (“pulpitis”[MeSH Terms] OR “pulpitis”[All Fields]) AND (“mandibular nerve”[MeSH Terms] OR (“mandibular”[All Fields] AND “nerve”[All Fields]) OR “mandibular nerve”[All Fields] OR (“inferior”[All Fields] AND “alveolar”[All Fields] AND “nerve”[All Fields]) OR “inferior alveolar nerve”[All Fields]) AND block[All Fields]).

The searches were limited to studies published in English from inception to April 5, 2018.

**Figure 1:**
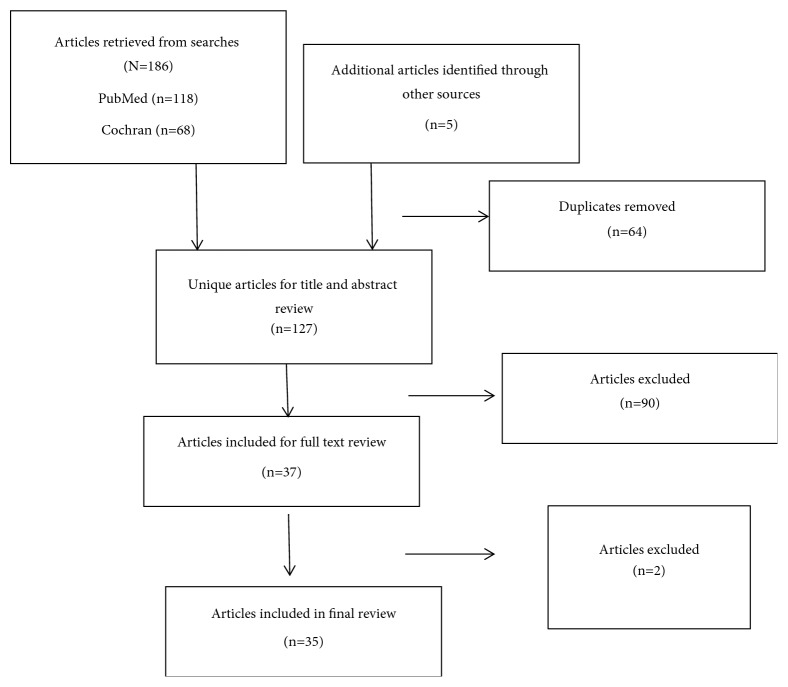
PRISMA Flow Chart.

**Figure 2:**
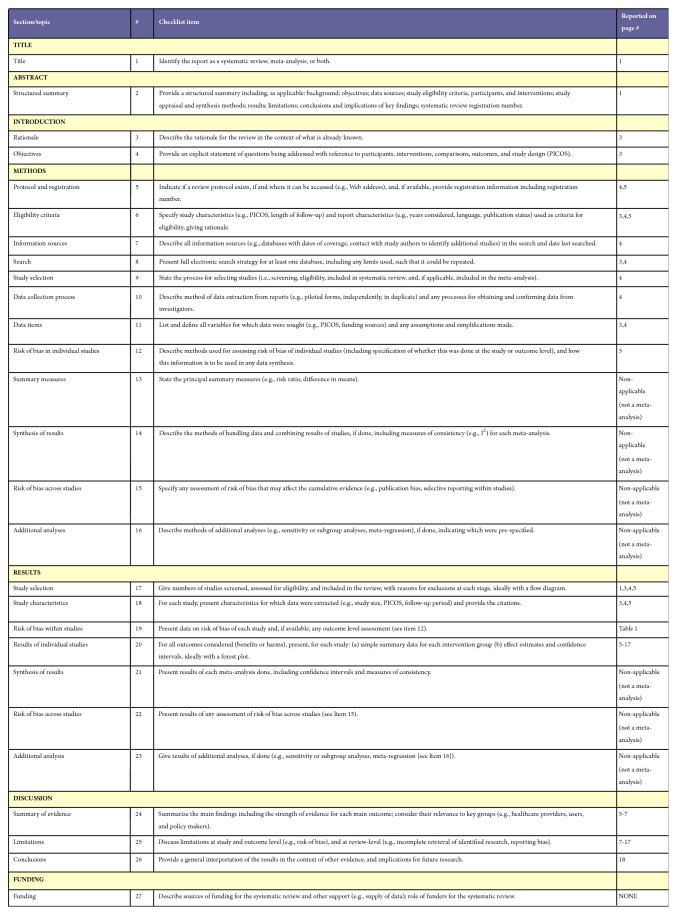
PRISMA checklist.

Only those articles in English and focusing on the effect of preoperative medication on the success of inferior alveolar nerve block were included in the core part of the review. The retrieved articles were individually read by 3 reviewers following which a meeting was performed in order to come to a final consensus about the manuscripts to be included. In case of discrepancy between authors, a common decision was made by mutual discussion. Overall, 37 studies were selected that met the required criteria (Table 1). The sought variables were the type of the study, anesthesia, number of patients included, type of premedication, presence of placebo, and final success rates. The obtained results were tabulated and possible biases were included in Table 2, such as the number and age range of study groups, the anesthetic solution used, the epinephrine ratio in anesthetics, and the dosage of administered premedications. The risks of bias were evaluated at the study level. The strategy used in the search is shown in Figure 1.  Figure 2 provides a PRISMA checklist for the items included.

This article will initially focus on the study methodologies with a commentary analysis and then summarize the points reported relevant to each group with a critical perspective and a final concluding remark.

### 2.2. Data Collection

Data collection was performed by 3 reviewers independently. Later, collected data were combined and a consensus was reached by excluding irrelevant papers or duplicates.

### 2.3. Risk of Bias Assessment

The Cochrane Collaboration tool [28] was used to evaluate the studies in terms of rick of bias. Seven criteria were selected to evaluate the studies. If the study provided detailed information, it was considered as low risk of bias whereas the manuscript was missing some important information (more than 2 of the selected criteria); it was considered as having a high risk. The paper was assigned as having moderate risk of bias when neither low nor high risk could be given. In case discrepancy existed between the reviewers, consensus was reached by detailed discussion.

## 3. Results

One hundred and eighteen papers were collected from PubMed whereas 68 papers were collected from Cochrane. Five additional articles were retrieved from other sources. Following the removal of 64 duplicates, 127 articles remained, 90 of which were excluded due to irrelevance, leaving 37 papers to be included for full-text review. Two papers were excluded one of which included patients younger than 18 years [29] and the other one delivered medication in an intrapulpal way [30]. Overall 35 studies were included (Table 2).

Twenty papers were determined to have a moderate level of bias whereas 12 were reported to have a high and 3 were reported to have a low level of evidence.

In the majority of the studies, age ranges of patients were established and indicated. One study did not report the age range of the patients included [4] whereas 1 study only reported that the age of the patients was above 18 [31].

Some studies preferred a wider age range when selecting patients to be included, from 18 until 65 or 72 [7, 20, 32–36]. On the other hand, a smaller age range was used in other studies by restricting the age limits to patients who are approximately 18-50 [37–42].

Although differences exist between the studies in terms of inclusion of age-groups, studies evaluating the pain perception of patients showed no difference in pain levels related to age or gender [43]. On the other hand, the anxiety levels of the patients were not taken into consideration in most studies as a factor which might have an impact on sensation and perception of pain. One study standardized anxiety levels by using Corah's dental anxiety scale [44] and some studies incorporating opioid analgesics also ranked the anxiety levels of patients [35], as well as some incorporating benzodiazepines [33, 40, 41]. Significant correlations were found between pain levels and dental anxiety in previous studies [45] leading us to assume that a preliminary anxiety testing would be beneficial before these types of studies to better standardize the samples. More valid conclusions can be obtained if patients with high anxiety levels are excluded as the impact of fear and apprehension may conceal the actual impact of medication on the overall success of local anesthesia [46].

When the studies pertaining to the topic are evaluated, there is no standardization with respect to the anesthetic solution used, though in the majority of studies 2% lidocaine has been selected as the anesthetic to be administered. In studies using lidocaine, some preferred the use of 1:80000 epinephrine [31, 32, 34, 47] whereas others used 1:100000 epinephrine as the vasoconstrictor [7, 32–35, 38, 40, 41, 44, 48–50]. In some studies, 1:200000 epinephrine was selected along with Lidocaine [19, 25, 37, 39, 51] whereas articaine with 1:100000 epinephrine was the selected anesthetic in one study [36] along with tramadol injection. One study used mepivacaine with 1:100000 epinephrine [20] and one used 2% mepivacaine with 1: 100 000 epinephrine [42].

In terms of initial diagnosis for admission to the study, participants who experienced typical symptoms of acute irreversible pulpitis and those with prolonged response to cold testing were included in the majority of the investigations. On the other hand, in some studies [32, 34], patients with prolonged response to cold were selected rather than spontaneous pain.

In the majority of studies, the effectiveness of anesthesia was tested based on one test, either lip numbness, electric pulp tester, or the presence of pain during endodontic access cavity preparation [19, 32, 34, 39].

De Pedro-Munoz and Mena-Alvarez [36] suggested the use of multiple diagnostic tests to confirm the establishment of anesthesia based on the well-known fact that negative response to lip numbness or to cold or electric tests does not guarantee pulp anesthesia when performing access cavity preparation. Thus, it appears beneficial to confirm the establishment of anesthesia by multiple assessments beginning with cold testing until endodontic access preparation.

## 4. Discussion

### 4.1. Lidocaine Articaine

Although lidocaine was used in the majority of the studies, articaine is also one of the most reputable anesthetics which has been approved as a safe solution. Its chemical composition shows that it contains a thiophene ring instead of a benzene ring and unlike other amide group local anesthetics; it has an extra ester linkage in the articaine molecule [52]. The clinical efficacy of articaine and prilocaine has been shown to be favorable and comparable to other local anesthetics. Although complications such as paresthesia have been reported to be higher with articaine in some studies [53] this parameter is regarded as questionable and a very rare clinical event [54].

A search of the literature reveals that articaine has been extensively studied in terms of anesthetic efficacy and lidocaine and articaine have been compared in a variety of studies in terms of their efficacy in irreversible pulpitis [55–60]. In general, articaine was found to be more efficient in mandibular infiltrations rather than nerve blocks [5]. On the other hand, it is logical to assume that this aspect of articaine should not be overlooked and further studies with premedication incorporating articaine as a supplementary infiltrative anesthesia can be conducted to find out whether it has any additional benefit on the achievement of successful anesthesia in premedicated patients.

### 4.2. Preoperative Pain

Parirokh et al. (2010) speculated that the utilization of premedication in patients with spontaneous pain was nonbeneficial due to the fact that the prostaglandins have already been released and cause the formation of TTX-resistant receptors responsible of anesthetic failure. They added that premedication can only be helpful in the enhancement of anesthetic effect in patients who have prolonged response to cold without any spontaneous pain. This raises a question in terms of inclusion criteria when testing the efficacy of premedication on IANB in patients undergoing irreversible pulpitis [34].

#### 4.2.1. Premedications

Table 2 summarizes all studies on the effect of premedication on the success of IANB. In this article, the studies were evaluated by classifying the type of premedication selected to enhance the anesthetic effect to provide a better understanding of the influence of each type of medication used.

### 4.3. Nonsteroidal Anti-Inflammatory Drugs (NSAIDs)

Nonsteroidal anti-inflammatory drugs (NSAIDs) are the most commonly administered group of analgesics used in dentistry [61]. Their mode of action is to block the cyclooxygenase enzyme, thus lowering the levels of prostaglandins produced in the arachidonic acid pathway. Specifically, PG2 is the prostaglandin that has been shown to be effective in the nociceptor neurons by sensitizing the transmembrane voltage-gated sodium channels (VGSCs). The result of such an effect is high susceptibility of these channels to major inflammatory mediators, histamine and bradykinin. Painful episodes and hyperalgesia ensue with such an interaction and the effect of administered anesthetics is also hampered [62]. Consequently, prostaglandin suppression has been suggested as very important for the alleviation of painful symptoms [63].

The effect of premedication with NSAID has been widely studied and ibuprofen has specifically been preferred by either administering the medication alone or comparing it with other drugs [4, 19, 37, 47]. A recent meta-analysis on NSAIDs revealed that this group of medication can increase the efficacy of IANB; however, the anesthetic type, volume, or supplemental injections do not seem to have any effect. Thus, NSAIDs appear to be a group of drugs that deserve specific attention for facilitating the IANB anesthesia [64].

#### 4.3.1. Ibuprofen

Oleson et al. [44] reported that 800 mg. ibuprofen administered before IANB for mandibular teeth caused no statistically significant anesthetic efficacy compared to patients who did not receive any medication. They supported their findings by the fact that prostaglandins whose synthesis is inhibited by ibuprofen is only one group of the mediators released during inflammation among others such as serotonin and histamine. Consequently, the absence of this mediator only would not be sufficient to desensitize the resistant sodium channels that cause persistence of pain. Contrary to the findings of Oleson et al., Noguera-Gonzales et al. [20], who compared premedication with 600 mg ibuprofen with placebo, came to the conclusion that administration of ibuprofen caused a significant improvement in the efficacy of IANB. On the other hand, the difference in their study was the administration of mepivacaine instead of lidocaine as the local anesthetic. The authors supported the use of mepivacaine instead of lidocaine based on the suggestions by Hargreaves and Keiser [65] who indicated that utilization of lidocaine in inflamed areas is not favorable due to the reduced effect of the anesthetic on the mediators and the alteration of the lidocaine molecule by the acidic environment, preventing the transmission of the molecule through the cell membrane. The authors explained their preference of mepivacaine by the fact that this anesthetic is more resistant to ion trapping. Because of the limited number of studies using mepivacaine, further research is mandatory to assess the increase in anesthetic effect when premedication is used in combination with mepivacaine as an anesthetic solution.

#### 4.3.2. Ibuprofen and Acetaminophen

Acetaminophen is another popular option in the control of pain and preferred by many dental practitioners. Despite the fact that the action mechanism of acetaminophen is not clarified, it is thought to interfere with inflammation by reducing prostaglandin synthesis (presumably PGF2) [32]. It is also believed to affect pain transmission by having direct impact on an unknown site of the brain and interact with both cannabinoid and serotoninergic pathways [48]. Because these peripheral and central action mechanisms differ from that of ibuprofen, the combination of ibuprofen and acetaminophen has been proposed as an alternative option as acetaminophen may have the potential to compensate for the effect ibuprofen is unable to do alone. It has been stated that the effect of IANB anesthesia can be enhanced by the combined use of acetaminophen and ibuprofen [37, 48].

Studies incorporated both ibuprofen and acetaminophen and either compared the effect of these medications when used alone or evaluated the outcome when they were combined. Modaresi et al. [4] evaluated the efficacy of the administration of acetaminophen+codeine and ibuprofen in symptomatic patients one hour before the delivery of anesthesia and concluded that preoperative administration of ibuprofen, if not contraindicated, can be a drug of choice 1 hour before local anesthesia to facilitate the success. On the other hand, Ianiro et al. [32] compared acetaminophen alone or in combination with ibuprofen and determined no significant differences despite a trend toward better success in the medicated groups. Simpson et al. [48] reported similar findings and reported that the combination dose of 800 mg ibuprofen and 1000 mg acetaminophen given 45 minutes before administration of the IAN block did not cause any significant increase in anesthetic efficacy. Nevertheless, similar to the findings of Ianiro et al., there was a trend toward better clinical success with the medications compared to the placebo group. Another study supporting the use of ibuprofen over acetaminophen was by Madani et al. who included ibuprofen, gelofen (another propionic acid derivative similar to ibuprofen), and acetaminophen in their study groups. Premedication with both ibuprofen and gelofen significantly affected the anesthesia quality in mandibular molars contrary to acetaminophen which resulted in no significant improvement. They compared their results to those of Parirokh et al. [34] who also reported favorable findings with ibuprofen. On the other hand, an interesting finding was that, despite the inclusion of patients exhibiting different symptoms, both studies reported favorable outcomes. Although patients with spontaneous pain exhibiting signs of irreversible pulpitis were used in the study by Madani et al., ibuprofen and gelofen seemed like suitable choices for medication [29].

#### 4.3.3. Ibuprofen, Other NSAID and Corticosteroids

Ibuprofen has not only been compared with other NSAID in terms of enhancing anesthetic success but different NSAIDs have also been compared with each other. In one study [34], ibuprofen was compared with indomethacin, another NSAID which has strong anti-inflammatory effects mostly used for muscular and joint pain. Both medications were found to significantly increase the success rates of IANB in symptomatic teeth with irreversible pulpitis. Despite the similar actions of both medications, the authors favored the use of ibuprofen instead of indomethacin in endodontic cases due to the fact that it has fewer side effects compared to the latter.

In another study [37], ibuprofen was compared with another nonsteroidal anti-inflammatory drug ketorolac (KETO), in the arylalkanoic acid group. Ketorolac is a pyrrolo-pyrrole derivative, as effective as morphine or meperidine for pain relief. It has been proposed that the mechanism of action of KETO is the inhibition of conduction of C fibers, which are more resistant to local anesthesia compared to A-delta fibers [25]. The authors reported that preoperative administration of ibuprofen or ketorolac had no significant effect on success of IANB in patients with irreversible pulpitis. The authors attributed the lack of success of premedication to the fact that the nociceptors were already activated by the inflammatory reaction.

Jena and Shaskirek [7] incorporated different combinations of NSAID to be compared to ibuprofen alone. They not only used ibuprofen alone but also administered ketorolac, etodolac+ paracetamol, and aceclofenac+paracetamol. The authors concluded that IANB alone is not sufficient to obtain thorough anesthesia in mandibular symptomatic teeth and additional supplementary anesthesia is definitely needed. Furthermore, they reported that administration of ketorolac 10 mg, 45 minutes prior to intervention improved success. They found no significant success with the other evaluated groups.

Lornoxicam was another NSAID compared with ibuprofen in terms of improving IANB. Similar to ketorolac, lornoxicam was also thought to inhibit the conduction of C fibers [66]. Jalil et al. [66] found no significant difference in the enhancement of IANB when premedication with ibuprofen and lornoxicam was compared. There was statistically no significant difference in the success rate of local anesthesia in patients with acute irreversible pulpitis of mandibular posterior teeth with premedication of ibuprofen and lornoxicam. The authors drew attention to individual differences in perception of pain as well as the difference in the microbial flora in the root canal system leading patients to display varying degrees of response to stimuli.

Few studies compared ibuprofen's effect with corticosteroids, one of which is dexamethasone.

Glucocorticosteroids exert their effect by decreasing vasodilation, leukocyte migration and inhibiting arachidonic acid formation. This results in the blocking of COX and lipoxygenase pathways and the synthesis of prostaglandins and leukotrienes [31].

In a study comparing ibuprofen with dexamethasone, premedication with dexamethasone was found to increase the success rate of an IANB in mandibular molars with asymptomatic irreversible pulpitis whereas ibuprofen had no statistically significant effect compared to the control group [31]. The authors compared their study with previous investigations by Moderasi et al. [4] where ibuprofen was found to have a significant effect on the success of IANB and by Aggarwal et al. [39] where supplementary dexamethasone injection resulted in no significant difference. They attributed the differences between the studies to the study design such as the inclusion of patients and the difference in dosages of medications. They also explained the superiority of dexamethasone administration over ibuprofen with the mode of action of the drugs. Their interpretation of the findings was the fact that glucocorticosteroids cause blocking of the COX and lipoxygenase pathways whereas nonsteroidal anti-inflammatory drugs inhibit the COX2 pathway. Nevertheless, it is apparent from these studies that no matter which type of medication is used, standardization with respect to dosage as well as initial diagnosis is essential in these types of studies to obtain more reliable results. A recent study also compared the use of ibuprofen with dexamethasone and concluded that both medications increased the success rate of IANB; however there were no significant differences between the two [67]. Their findings on the success of ibuprofen were contradictory to those reported by Aggarwal et al. [37], Oleson et al. [44], and Shahi et al. [31] where ibuprofen was found to have no significant effect. They explained these findings with the patient selection in their study where inclusion criteria were the presence of prolonged pain in response to cold contrary to the others where spontaneous pain was the selection criteria. They supported their findings by the comments previously made by Parirokh et al. [34] who stated that there is a high level of previously released prostaglandins in cases of spontaneous pain, resulting in lowered ability of premedication to be effective.

Another nonsteroidal anti-inflammatory drug compared with ibuprofen was Meloxicam, a drug used in the treatment of osteoarthritis and rheumatoid arthritis and with milder effects on the gastrointestinal tract due to its activity on the cyclooxygenase 2 (COX-2) system [50, 68]. This was probably one of the reasons why it was compared with ibuprofen as it would provide an additional benefit in patients with gastrointestinal problems. Although one drawback of COX-2 inhibitors such as rofecoxib and celecoxib was reported as the elevation of myocardial infarction and stroke risk, Meloxicam has been reported to be safer in that respect [60]. Although both ibuprofen and Meloxicam yielded more successful results compared to the control group, no significant difference was noted between the two medications. The authors suggested that different dosages of this medication should be studied to make a better clarification.

#### 4.3.4. Other NSAIDs

Although ibuprofen has been the major focus of attention, different classes of NSAID have also been compared in terms of their ability to facilitate the effect of IANB block. One of these is lornoxicam, a drug in the oxicam class of NSAIDs and which is prescribed for osteoarthritis, rheumatoid arthritis, acute lumbar-sciatica pain and for postoperative pain management [19]. Prassana et al. [19] compared the efficacy of lornoxicam (LNX) with that of diclofenac (DP), another NSAID, mainly used for the treatment of pain and inflammation related with rheumatic disorders. They concluded that preoperative administration of LNX used preoperatively had a significant influence on improving the effectiveness of IANB in irreversible pulpitis cases whereas results with DP was not significant compared to the control group where placebo was used. They explained the successful response by lornoxicam with transient receptor potential vanilloid channels associated with pain signaling and thermos-reception. They indicated that specifically TRPV1 is responsible in hyperalgesia and allodynia as well as mediation of pain of pulpal origin. Although statistically not different than diclofenac, the higher success rate obtained by LNX was attributed to its ability to better inhibit TRPV channels.

A promising result was obtained using acclofenac, administered 45 minutes before IANB. It was found to significantly increase the anesthetic success rates in patients with irreversible pulpitis [49].

Aggarwal et al. [39] determined that buccal infiltration of articaine and articaine plus Ketorolac significantly increased the success rate of IANB. On the other hand, where supplementary infiltration was made using dexamethasone, no significant difference was found compared to the control group. Akhlaghi et al. [69] reported results in favor of buccal injection with Ketorolac after IANB and suggested that this approach significantly affected the quality of anesthesia. Their results were contradictory to the results by Aggarwal et al. [37] who determined that oral premedication with ibuprofen or ketorolac did not significantly increase the success rate of IANB injections. One drawback associated with the infiltrative use of ketorolac was pain experience by patients during administration. To eliminate the possibility of such unpleasant sensation by the patients, the authors initially delivered some local anesthetic in the area of the injection before administering the ketorolac, resulting in no pain experienced by the participating patients.

Wali et al. [70] compared 3 different types of NSAID which are piroxicam, diclofenac, potassium and naproxen sodium which are acknowledged as fast acting analgesics capable of reducing pain within a period of 15-30 minutes. Their results showed that premedication in general was beneficial in enhancing the anesthetic effect though piroxicam seemed to yield significantly higher success rates compared to naproxen sodium. The authors also criticized the study design by indicating that a higher number of patients need to be incorporated to obtain more valid results.

Ketorolac was found to be significantly effective in enhancing anesthetic efficacy in a study by Yadav et al. [47]. However, this study not only focused on premedication but the type of anesthesia as well. The authors concluded that ketorolac premedication followed by an articaine IANB with buccal and lingual infiltrations caused significantly higher success compared to an articaine IANB and ketorolac, lidocaine IANB and Ketorolac, and a lidocaine IANB and infiltration. Therefore, it is difficult to make a direct extrapolation on the effect of premedication and make a definite statement as whether it was the premedication or the type of anesthesia that specifically influenced the overall result. Nevertheless, the study is promising to bring a recommendation to the delivery and selection of anesthesia in teeth with irreversible pulpitis.

Saha et al. [25], on the other hand, compared diclofenac and ketorolac, two NSAIDs in the arylalkanoic acid group and reported results in favor of oral premedication with 10 mg ketorolac. They indicated that ketorolac resulted in significantly higher success in IANB in patients with irreversible pulpitis compared to 50 mg. diclofenac. These results were in compliance with those reported by Jena and Shaskirek [7]; however contradictory to those reported by Aggarwal et al. [36, 37] who reported no significant improvement due to its use.

### 4.4. Opioid Analgesics

Opioids exert their analgesic effect by interacting with opioid receptors located in the terminal regions of nociceptors. They bind to receptors which are upregulated due to tissue injury and analgesia is obtained [35].

Hydrocodone is also one of the opioids that has been investigated in terms of enhancing anesthetic efficacy. In one study, acetaminophen [35] was investigated when used in combination with hydrocodone in terms of increasing success of IANB anesthesia in symptomatic teeth. The authors found no difference in terms of increase of anesthetic efficacy when combination dose of 1000 mg acetaminophen/10 mg hydrocodone was administered 60 minutes before the administration of the IANB in patients with irreversible pulpitis. They proposed that opioids may not be effective during acute pain at a region where inflammation occurred previously. Furthermore, they drew attention to individual differences in terms of response to opioid analgesics and indicated that some individuals may require higher doses of opioids to obtain a beneficial effect and some are rapid metabolizers. Another finding of the study was that regardless of being medicated or not, majority of the patients were satisfied with the experience and it is not only the medication that plays a role in patient comfort but the general attitude of the practitioner as well as the emergency procedure itself which gives the patient the hope that pain will subside. The euphoric side-effect of hydrocodone was also presumed to be a factor leading to patients' reporting higher mean-satisfaction ratings compared to the control group. However, side-effects of opioids such as sleepiness and nausea should always be considered before prescribing to patients and premedication with opioids should be thought as an option only when benefits outweigh the disadvantages.

Tramadol is another opioid analgesic evaluated for its efficacy in enhancing anesthetic success. It is reported to bind weakly to *μ* receptors and has an inhibitory effect on the reuptake of serotonin and noradrenaline [71, 72]. It has been suggested by some authors that tramadol exerts anesthetic effect which is similar to lidocaine [73] and can even be used in some surgical procedures as an anesthetic solution [74].

Rodriguez-Wong et al. [42] administered the combination of mepivacaine with tramadol and determined similar success rates with mepivacaine 2% with 1: 100 000 epinephrine during IANB. On the other hand, de Pedro-Munoz and Mena-Alvarez [36] determined significantly better success rates with tramadol when used as a local injection during endodontic access cavity preparation. The authors compared their results with that of Rodriguez-Wong et al. [42] by the mode of delivery of tramadol. They drew attention to the fact that opioids show their effect better under inflammatory circumstances and hyperalgesia and their administration to the area with nerve damage is more successful than application at a distance. Furthermore, as a secondary finding, men had an anesthetic effect of longer duration compared to women. Nevertheless, the authors reported the reduced number of samples as a limiting factor and advocated the necessity of larger sample sizes to make more valid conclusions.

A favorable result with tramadol was found by Mahajan et al. [51] where this opioid was compared with ibuprofen (600mg), and combination of ibuprofen (400 mg) + acetaminophen. Although tramadol significantly increased the anesthetic efficacy compared to the other medications tested, the authors drew attention to the fact that it decreases the seizure threshold and excluded epileptic patients in which the administration of the drug is contraindicated.

Meperidine is an analgesic, sedative, and antispasmodic agent, also known as pethidine or Demerol. It has also been studied in terms of its efficacy to increase success of IANB [75]. Tough the exact mechanism of action is not clarified, it has been reported that meperidine binds to opioid specific receptors and also has an anesthetic effect [75]. Bigby et al. [75] combined meperidine with lidocaine solution and administered the combination in a conventional IANB; however, no improvement was observed in the success rate in comparison to standard lidocaine solution. The authors performed the study based on some favorable results reported by some authors who suggested that there was synergism between meperidine and lidocaine [76]. The finding that there is no favorable improvement in anesthetic efficacy when 2 medications were combined was explained by the removal of lidocaine from the injection site as well as the dilution of lidocaine by the addition of meperidine.

### 4.5. Benzodiazepines

Conscious sedation is a methodology that is used in dentistry specifically for patients with high anxiety levels and benzodiazepines are the most commonly used sedatives due to their pain reducing ability and safety. Benzodiazepines stimulate* Gamma-Amino-Butyric* Acid-A (GABAA) receptors in dorsal horn of spinal cord and act against hyperalgesia by reducing the pain-related anxiety. They stimulate the release of endogenous opioids such as encephalins in central nervous system areas that take part in pain processing [77].

Triazolam is an anxiolytic agent that is advocated to be used in endodontic patients. Lindemann et al. [33] determined that 0.25 mg. of triazolam used sublingually did not increase the effectiveness of IANB in patients with irreversible pulpitis. Despite this result, the authors drew attention to the significance of anxiety and fear reduction and indicated that it may help endodontic treatment to be more acceptable by the patients.

Alprazolam is one the most frequently used benzodiazepines used for the elimination of abnormal excitement in the brain and treatment of anxiety disorders [40]. Alprazolam was also evaluated in terms of its efficacy to enhance the anesthesia quality of IANB in symptomatic teeth with irreversible pulpitis. In the first study on this topic, preoperative oral administration of 0.5 mg of alprazolam was found to have no effect on the improvement of success of IANB [40] on the other hand, Shetkar et al. [41] used different anesthesia techniques along with different types of premedication and found results in favor of alprazolam; however, they combined alprazolam with diclofenac potassium, a NSAID. They also determined that Gow Gates (GG) nerve block along with premedication is the best method for effective pain management of acute pain in irreversible pulpitis.

It can be assumed based on the performed studies that benzodiazepines alone do not appear to exhibit an increasing effect of IANB in patients undergoing irreversible pulpitis. Combination of benzodiazepines with other analgesics such as NSAID appear to be beneficial. Nevertheless, they seem to be a good option for the management and anxiety reduction of apprehensive patients provided that a deep profound anesthesia is also achieved. Considering the limited number of research involving this group of medication, further investigations are definitely warranted.

### 4.6. Nitrous Oxide and other Sedatives

Nitrous oxide, the most commonly used inhalation anesthetic in dentistry, was first evaluated in terms of enhancing anesthetic effect of IANB by Stanley et al. [78] resulting in a significantly beneficial effect. The authors reported that nitrous oxide targets both opiate receptors and NMDA receptors to provide analgesia. They selected a concentration of 30%–50% nitrous oxide in their study and reported that concentrations such as 70% would cause more side effects such as nausea and vomiting. Stentz et al. [79] administered intranasal ketorolac before nitrous oxide sedation; however, this approach failed to make a significant improvement on IANB and the authors concluded that supplemental anesthesia would still be required.

Ketamine, a derivative of phencyclidine and mainly used for starting and maintaining anesthesia. It induces a trancelike state while providing pain relief, sedation, and memory loss. Other uses include for chronic pain and for sedation in intensive care. It has been evaluated in terms of its potential to facilitate anesthetic effect. Ketamine has been reported to reduce pain by interacting with N-methyl D-aspartate (NMDA) receptors, opioid receptors, monoaminergic receptors, muscarinic receptors, and calcium and sodium ion channels. It also has the potential to cause nerve block similar to local anesthetics [38]. Oral administration of ketamine was shown to significantly reduce the number of cartridges used for IANB in patients with irreversible pulpitis and post-operative pain was significantly lower [38]. The limited number of research on this group of drugs necessitates further supporting research. Nevertheless, its beneficial effect on anesthesia and post-operative pain even in low dosages renders the medication to be an alternative in the management of patients experiencing symptoms of irreversible pulpitis.

Another study where ketamine was investigated was by Sakhaeimanesh et al. [80]. Instead of oral administration, the authors added ketamine to articaine anesthetic solution. However, adding 0.4 mL 50 mg/mL ketamine had no favorable effect on the efficacy of IANB for posterior mandibular teeth with symptomatic irreversible pulpitis. The authors attributed this result to the different onsets of action of ketamine and articaine as well as the limited sample size.

### 4.7. Hyperosmolar Solutions

Mannitol, an osmotic diuretic capable of inducing diuresis, also has the potential to open the perineural membrane to enhance the penetration of macromolecules [18]. For this reason, it was evaluated in terms of its effect on enhancing the effect of local anesthesia when administered along with a local anesthetic. Kreimer et al.[18] reported that when mannitol (0.5 mol/L) was added to lidocaine, statistically significant success rates were obtained though the success rate did not result in predictable pulpal anesthesia.

### 4.8. Antihypertensives and Magnesium Sulfate

Clonidine is a selective alpha-2 adrenoceptor agonist having central and peripheral actions. It is used as an antihypertensive agent and has also been shown to facilitate the effect of local anesthesia. The medication not only reduces blood pressure but it also causes sedation and analgesia without any cardio toxic effect; therefore, it has also been used as an alternative vasoconstrictor instead of epinephrine [81]. Shadmehr et al. [81] compared the efficacy of lidocaine with clonidine and lidocaine with epinephrine in IANB block and concluded that, in mandibular molars with irreversible pulpitis, clonidine+lidocaine combination significantly improve the success of IANB. Furthermore, the combination lead to an insignificant decrease in heart rate and blood pressure compared to the significant increase of these parameters in the lidocaine+epinephrine group, which was regarded as one of the advantages. Additionally, because of the sedation effect of clonidine, experience of pain would decrease due to the reduction in anxiety levels [81].

Magnesium sulfate, an adjunct to increase anesthetic effect in various branches of medicine, was also among the medications tested in terms of increasing anesthetic efficacy. Shetty et al. [82] administered either 1 mL magnesium sulfate USP 50% or distilled water prior to conventional IANB and concluded that preoperative administration of 1 mL. magnesium sulfate significantly increased the success of IANB. The authors indicated that magnesium sulfate is a promising agent as it is both inexpensive and safe and the medication has a good NMDA receptor antagonism as well as calcium channel blocking effect. They advocated these favorable properties warrant further research to be conducted by using different dosages before it can be recommended for routine use.

### 4.9. Systematic Reviews and Meta-Analysis

A search of the literature reveals limited number of studies fullfill the eligibility criteria (Table 1) and reviews pertaining to the topic of the effect of medication on the success of IANB. A meta-analysis on the influence on NSAID showed promising results for this group of medications but drew attention to the necessity of further research [83] (Li et al. 2012). Similarly, Lapidus et al. [84] stated that existing evidence for the use of oral NSAIDs, particularly 600 mg. ibuprofen was moderate and limited. Tupyota et al. [85] on the other hand made more definite conclusions and indicated that both the increase in the volume of the anesthetic along with the administration of NSAIDs is a predictable means of achieving successful anesthesia for pain control in mandibular teeth with irreversible pulpitis. Corbella et al. [64] supported the use of premedication with anti-inflammatory drugs to facilitate the anesthetic effect of IANB. On the other hand, the authors concluded that the type, volume and supplemental buccal injection did not have any influence. A recent meta-analysis made a different conclusion compared to others and favored the use of oral premedication with dexamethasone, NSAIDs or tramadol; yet drew attention to the necessity of more trials [86].

## 5. Conclusions

This article intended to make a general overview of studies performed on premedication for the stimulation of IANB anesthesia in patients with irreversible pulpitis. As understood, variation exists with respect to methodology used, making it impossible to make definite conclusions. Standardization appears necessary with respect to sample size, initial diagnosis and dosage as well as factors such as anxiety levels and degree and duration of pain.

Within the limitations of this review, five major strategies that can be proposed to facilitate the success of IANB anesthesia can be summarized as follows: (1) Articaine can be preferred rather than lidocaine for mandibular infiltrative anesthesia. (2) Mepivacaine can be prefered rather than lidocain. (3) Ibuprofen and some other NSAIDs appear to be medications that may contribute to the overall success of IANB rather than Acetaminophen.(4) Acetaminophen can be used for premedication if NSAIs are contraindicated. (5) Opioids may be preferred as analgesic, sedative and antispasmolytic agents provided that they are not administered in patients in whom they are contraindicated.(6) Nitrous oxide and other sedatives may be preferred in severely apprehensive patients.(7) Oral administration of ketamine can be used to reduce the number of cartridges used for IANB in patients with irreversible pulpitis and post-operative pain was significantly lower. (8) Addition of Clonidine, a selective alpha-2 adrenoceptor agonist having central and peripheral actions to lidocaine may improve success rate of IANB

Though some medications or combination of medications appear to be promising, further supporting research will help highlight this significant topic which yet requires more clarification.

## Figures and Tables

**Table 1:** Eligibility criteria for the papers to be included.

Randomized clinical trials where subjects older than 18 years were included
Patients with signs and symptoms of irreversible pulpitis and who required endodontic treatment were included
Patients had to be administered an inferior alveolar nerve block anesthesia using contemporary local anesthetics on the market
Trials where different types of premedication were applied were included.
Power analysis should be made
The type and dosage of anesthesia should be mentioned
The type of pain scoring should be expressed
The success criteria should be clearly defined

**Table 2:** Studies on the success of IANB using premedication by classification with drug type.

Author year	Study design	Power analysis	Corah dental anxiety	Initial pain	Anesthetic	Supplementary injection	No. of patients evaluated teeth-success rate	M/F	Age	Medications used	Success criteria	Results	Risk of bias
**IBUPROFEN**													

Oleson *et al.* 2010	Prospective Double-blind Randomized (no explanation) Placebo-controlled	no	yes	Heft-parker vas	2% lidocaine with 1:100,000 epinephrine (0.9 mL for the long buccal injection)		100 Mandibular posterior teeth (molar or premolar); No subjects were eliminated as a result of lack of lip numbness after 15 min	45 M/ 55 F	32±8;33±12	Ibuprofen (800 mg) Placebo	Success=no or mild pain (heft-parker VAS) on pulpal access or instrumentation	No statistical significance	medium

Noguera-Gonzales *et al.* 2013	Double-blind Randomized (The patients were assigned sequential numbers in the order of their enrolment and received their allocated treatment according to a randomization schedule designed previously.) Placebo-controlled	yes	no	Heft-parker vas	2% mepivacaine with 1:100 000 epinephrine.		50	18 M/ 32 F	18-68	Ibuprofen (600 mg) Placebo	Failure=no lip numbness after 15 min or painful response to cold or painful response to endodontic access; heft-parker Vas only at the beginning	Significant improvement in the efficacy of IANB with preoperative administration of Ibuprofen.	medium

**IBUPROFEN AND ACETAMINOPHEN**													

Modaresi *et al* 2006	Randomized (no explanation) Placebo-controlled Double-blind	no	no	Ept	2% lidocaine with 1:80 000 epinephrine (If no subjective signs of anesthesia, consisting of lower lip and tongue tip numbness, appeared within the time interval, readministration)		60	no	no	Ibuprofen (200 mg) Acetaminophen (300 mg)+ codeine Placebo	Electric pulp tester-lower tooth sensitivity level	Ibuprofen giving higher success compared to acetaminophen+codeine	high

Ianiro *et al*. 2007	Randomized (medications were assigned random numbers, patient draw a bottle from a box) Placebo-controlled Double-blind	yes	no	vas	2% lidocaine with 1:100,000 epinephrine,		40 Mandibular posterior teeth	16 M/ 24 F	19-72	Acetaminophen (1000 mg) Acetaminophen (1000 mg) + Ibuprofen (600 mg) Placebo	Failure=Sensitivity to cold 15 min later or sensitivity to the access procedure; Vas only at the beginning	No significant difference between the groups; however, a trend toward higher success in the medication groups	medium

Simpson *et al*. 2011	Prospective Double-blind Randomized (no explanation) Placebo-controlled	no	yes	Heft-parker vas	2% lidocaine with 1:100,000 epinephrine (0.9 mL for the long buccal injection)		100 Mandibular posterior teeth (molar or premolar)	36 M/ 64 F	Means 32 and 33	Ibuprofen + acetaminophen (800 mg/1000 mg) Placebo	Success=no or mild pain (heft-parker VAS) on access, clean and shape	No significant difference	medium

**IBUPROFEN, OTHER NON-STEROIDAL ANTI-INFLAMMATORY DRUGS AND CORTICOSTEROIDS**													

Parirokh *et al.* 2010	prospective Randomized (patient chooses an opaque envelope) Placebo-controlled Double-blind	yes	no	Heft-parker vas Significantly higher preoperative vas scores for indomethacin	2% lidocaine with 1:80000 epinephrine		150	71 M/ 79 F	18-64	Ibuprofen (600 mg) Indomethacin (75 mg) Placebo	Success=no or mild pain (heft-parker VAS) on pulpal access (within dentin, entering the chamber, file inserted into canal); failure=sensitivity to cold anytime after ianb	Significant improvement in IANB with preoperative administration of both Ibuprofen and Indomethacin.	medium

Aggarwal *et al.* 2010	Prospective Double-blind Randomized (with a linear congruential generator) Placebo-controlled	yes	no	Heft-parker vas with no mm	2% lidocaine with 1:200,000 epinephrine		69 (3 patients excluded unsuccessful anesthesia)	36 M/33 F	21-38	Ibuprofen (300 mg) Ketorolac (10 mg) Placebo	Heft-parker Vas in case of pain during endodontic treatment (success=no pain or weak/mild pain during Access preparation and instrumentation; The millimeter marks were removed from the VAS	No significant difference	medium

Aggarwal *et al.* 2011	Prospective Randomized (simple random generator) Double-blind Control (no supplemental injection)	yes	no	Heft-parker vas with no mm	2% lidocaine with 1:200,000 epinephrine		94 (2 patients excluded unsuccessful anesthesia 2 patients excluded for severe transient injection pain- ketorolac tromethamine)	45 M/ 49 F	24-36	supplemental buccal infiltration of 4% articaine with 1:100,000 ephinephrine; supplemental buccal infiltration of 1 mL/4 mg of dexamethasone; supplemental buccal infiltration of 1 mL/30 mg of ketorolac tromethamine preceeded by 0.9 mL of 4% articaine infiltration-10 min.	Heft-parker Vas in case of pain during endodontic treatment (success=no pain or weak/mild pain during Access preparation and instrumentation; The millimeter marks were removed from the VAS	Articaine and articaine+ketorolac significantly increased success rate	medium

Jena &Shashirekha 2013	Prospective Randomized (no explanation) Double-blind Placebo-controlled	no	no	Heft-parker vas	2% lignocaine with 1:100000 epinephrine		100	63 M/37 F	18-65	Ibuprofen (600 mg) Ketorolac (10 mg) Etodolac + Paracetamol (400 mg + 500 mg) aceclofenac + paracetamol (100 mg + 500 mg) placebo	Heft-parker Vas assessment during endodontic therapy	improvement with Ketorolac	high

Shahi *et al.* 2013	Double-blind Randomized (with a linear congruential generator) Placebo-controlled	no	no	vas	2% lidocaine with 1:80,000 epinephrine		165	86 M/ 79 F	Older than 18	Ibuprofen (400 mg), Dexamethasone (0.5 mg.) Placebo	Success=no or mild pain (VAS) on pulpal access or instrumentation	Significant improvement with dexamethasone	high

Jalil et al. 2014	Randomized (Lottery Method)	no	anxiety ratings were not significantly different	Vas	1.8ml of 2% Lidocaine with 1:100000 epinephrine.	pain was felt during access cavity preparation then outcome was recorded as failure and supplemental anaesthesia	120 104 *∗*55/60 *∗*49/60	68 M/ 49 F	18-50	*∗*Lornoxicam 8 mg -1 h before *∗*Ibuprofen 800 mg- 1 h before	Success = no pain during endodontic access and root canal instrumentation	No significant difference	medium

Bidar *et al.* 2017	Prospective Double-blind Randomized (balanced block randomization) Placebo-controlled	yes	no	Heft-parker vas	2% lidocaine with 1:80,000 epinephrine		78	30 M/46 F	20-60	Ibuprofen (400 mg.) Dexamethasone (4 mg) Placebo	Heft-parker Vas 15 min after iabn and in case of pain during treatment; success=no or mild pain at any stage during the endodontic procedure; failure=no lip numbness 15 min after injection)	Significant improvement with both Ibuprofen and Dexamethasone Significantly more effective (successful) than placebo	medium

Shantiaee *et al.* 2017	Double-blind Randomized (patient chooses an envelope) Parallel (Placebo and control groups)	no	no	Heft-parker vas EPT	2% lidocaine with 1:100,000 epinephrine		92	44 M/ 48 F	Means 29.26, 32.78, 31.7,32.22	Meloxicam (7.5 g.) Ibuprofen (600 mg) Placebo no medication	Success= no, or only mild, pain (VAS recordings) while preparing the access cavity or during initial instrumentation and no response to the EPT	Significant improvement with meloxicam and ibuprofen	high

**OTHER NON-STEROIDAL ANTI-INFLAMMATORY DRUGS**													

Prassana *et al*. 2011	Randomized (with a linear congruential generator) Double-blind Placebo-controlled	yes	no	Heft-parker vas	2% lidocaine with 1:200,000 epinephrine		114	55 M/ 59 F	21-40	Larnoxicam (8 mg) Diclofenac (50 mg) Placebo.	Failure=pain or sensitivity to cold and pain during access; Heft-parker Vas but The millimeter marks were removed from the VAS	Significant improvement with Larnoxicam; Significantly high post-injection vas scores in placebo	medium

Paul *et al.* 2011	Prospective Double-blind (no explanation) Randomized (no explanation) Placebo-controlled	no	yes	Heft-parker vas	2% lidocaine with 1:100000 epinephrine (0.9 mL for the long buccal injection)		40	23 M/ 17 F	Means 30.4 and 31.7 not younger than 18	Acclofenac (100 mg) Placebo	Heft-parker vas;success=no pain or weak/mild pain during Access preparation and instrumentation	Significant improvement with Acclofenac	medium

Wali et al. 2012	Randomized (no explanation) Placebo-controlled	no	no	-	1.8 mL of 2% lidocaine with 1:200,000 epinephrine	Access cavity preparation initiated experience pain-failure	80 42 *∗*%90 *∗*%75 *∗*%35 *∗*%10	42 M/ 38 F	adult	*∗*Piroxicam (20 mg) *∗*Diclofenavc potassium (50 mg) *∗*Naproxen sodium (550 mg) *∗*Placebo (drug becefol?) 1 h before	No pain during endodontic treatment	Significantly greater success with piroxicam compared to naproxen sodium and placebo; No difference between piroxicam and diclofenac potassium	high

Yadav *et al.* 2015	Prospective Double-blind Randomized (no explanationr) controlled	no	no	Heft-parker vas with no mm	4% articaine with 1:100,000 epinephrine 2% lidocaine with 1:80,000 epinephrine		150	78 M/ 72 F	20-35	(1) 0.9 mL BI and 0.9 mL LI with either articaine or lidocaine (2) Ketorolac (10 mg) (3) Ketorolac (10 mg) followed by BI and LI (0.9 mL each) with either articaine or lidocaine	Success= no, or only mild, pain (VAS recordings) while preparing the access cavity or during initial instrumentation	Significant improvement with Articaine + oral ketorolac premedication Significant improvement with Articaine +infiltration+ketorolac; Articaine+infiltrations better than lidocaine+infiltrations	high

Saha *et al.* 2016	Prospective Double-blind Randomized (with a linear congruential generator) Placebo-controlled	yes	no	Heft-parker vas with no mm	2% lidocaine with 1:200 000 epinephrine.		126	65 M/ 61 F	18-65	Ketorolac (KETO) Diclofenac Potassium (DP) Placebo	success=no pain during Access preparation and instrumentation	Significant improvement with Ketorolac; both for vas and success significant differences among three groups:ketrolac-diclofenac-placebo	medium

Akhlaghi et al. 2016	Prospective Double-blind (++) Randomized (random number table) Placebo-controlled	yes	no	Heft-parker vas with no mm, pain (visual analog scale >54) with prolonged response to cold testing (lingering pain for more than 45 seconds)	1.8 mL 4% articaine with 1:100,000 epinephrine (buccal infiltration of 0.9mL articaine immediately after the block injection)	Whenever an extra injection was applied because of severe pain at any stage, the patient's pain score was recorded, and that patient was excluded from the remainder of the study.-failure	40 11 *∗*%40 *∗*%15	16 M/ 24 F	18-65	After 5 minutes *∗*buccal infiltration of 30 mg/mL ketorolac tromethamine *∗*buccal infiltration of normal saline (Placebo)	Heft-parker vas; Success=absence of pain or only mild pain present during any of the stages of treatment (caries and dentin removal (CDR), access cavity preparation (ACP), and canal length measurements (CLM) stages)	ketorolac significantly increased the success rate	medium

**OPIOID ANALGESICS**													

Fullmer *et al*. 2014	Prospective Double-blind Randomized (random numbers identified the medications) Placebo-controlled	yes	yes	Heft-parker vas	2% lidocaine with 1:100,000 epinephrine (0.9 mL for the long buccal injection)		100 Mandibular posterior teeth (molar or premolar)	46 M/ 54 F	18-67	Acetaminophen (1000 mg) + Hydrocodone (10 mg) Placebo	Success=no or mild pain (heft-parker VAS) on pulpal access or instrumentation; second cartridge if nı lip numbness after 15 min-4in premedication and 5 in placebo group out of 100 patients)	No significant difference	low

Rodriguez-Wong *et al.* 2016	Randomized (computer generated randomization schedule) Double-blind Controlled	yes	no	Heft-parker vas (100 mm?)	1.3 mL of 2% mepivacaine with epinephrine 1 : 100 000 plus 0.5 mL of tramadol 50 mg mL 1 (experimental group) or 1.8 mL of 2% mepivacaine with epinephrine 1 : 100 000 (control group).		56	16 M/40 F	18-50	1.3 mL of 2% mepivacaine with 1 : 100 000 epinephrine + 0.5 mL tramadol 50 mg mL(experimental group) 1.8 mL of 2% mepivacaine with epinephrine 1 : 100 000 (control group).	Success= zero value on heft-parker vas (100 mm?) on all following steps: numbness of the lip, positive or negative cold test, asymptomatic management of dental hard tissues and access to dental pulp	No significant difference	medium

De-Pedro Munoz & Mena-Alvarez 2017	Randomized (nonprobabilistic sampling of consecutive cases, randomization software) Double-blind Placebo-controlled	Pilot study	no	vas	4% articaine with 1 : 100 000.epinephrine		42	equal	Mean 40.35 and 37.7	Tramadol (50 mg) mandibular infiltration and a placebo group	Success=2 consecutive negative response out of 3 to electric pulp tester at maximum and negative response to cold and no pain during access cavity preparation/instrumentation	Significant improvement with Tramadol; Tramadol significantly higher success during access cavity	high

Mahajan *et al.* 2017	Randomized (patient picks one of the slips with medication name on it from a box) Double-blind Placebo-controlled	no	no	Heft-parker vas	2% Lignocaine with 1:200,000 epinephrine		60	32 M/ 28 F	18-25	Ibuprofen (600mg), Tramadol (50 mg) and Ibuprofen (400 mg) + Acetaminophen Placebo	Failure=No Lip numbness after 15 min;success=Heft-parker vas no ormild pain during procedure	Significant improvement with Tradamol Success rate significantly higher compared to placebo	high

**BENZODIAZEPINES**													

Lindemann 2008	Prospective Double-blind Randomized (no explanation) Placebo-controlled	no	yes	Heft-parker vas	2% lidocaine with 1:100,000 epinephrine		58 Mandibular posterior teeth (molar or premolar)	34 M/ 24 F	18-62	Triazolam ( 0.25 MG) Placebo	success=Heft-parker vas no or mild on access or initial instrumentation	No significant difference	medium

Khademi et al. 2012	Prospective Double-blind Randomized (random number generator Placebo-controlled	no	yes	Heft-parker vas	2% lidocaine with 1:100,000 epinephrine		60	30 M/ 30 F	18-50	Alprazolam (0.5 mg) Placebo	success=Heft-parker vas no or mild on access cavity preparation and initial instrumentation	No significant difference	medium

Shetkar et al. 2016	Randomized (no explanation) Double-blind Placebo-controlled	yes	yes	Heft-parker vas	inferior alveolar, Vazirani-Akinosi, and Gow-Gates techniques 2% lidocaine with 1:100,000 epinephrine		180 (60 patients for each technique) 14 patients excluded unsuccessful anesthesia, *4 patients excluded for statistics*	96M/84 F	18-50	Placebo and Alprazolam (0.5 mg.)+ Diclofenac potassium (50 mg.) for all 3 types of anesthesia.	Heft-parker Vas following access cavity preparation or initial file placement	Significant improvement with Alprazolam combined with diclofenac potassium. for all 3 types of anesthesia.	low

**NITROUS OXIDE AND OTHER SEDATIVES**													

Kaviani et al. 2011	Randomized (on the day of treatment randomly assigned) Double-blind Placebo-controlled	no	no	no	2% lidocaine with 1/100000 epinephrine		36		15-45	Ketamine ( 10 mg) Placebo	Vas following treatment (positive response to pulp tester after 5-10 min-another cartridge injected IANB and the total number of cartridges recorded;during treatment in case of pain supplemental injections;questionnire for ibuprofen taken for the first 24 hours following treatment)	Significant improvement with ketamine	high

Stentz et al. 2018 +nitrous oxide	Prospective Double-blind (++) Randomized (6-digit random numbers) Placebo-controlled	no	no	Heft-parker vas, Spontaneous and greater than 54 mm-moderate or severe	3.6 mL 2% lidocaine with 1:100,000 epinephrine	topical anesthetic gel; molar teeth were given a buccal nerve block using 0.4 mL 2% lidocaine with 1:100,000 epinephrine for rubber dam clamp anesthesia. Failure-If the pain rating was moderate or greater during treatment, supplemental anesthesia was administered (buccal infiltration of articaine and/or intraosseous injections).	102 Mandibular posterior teeth (molar or premolar) 51 *∗*%54 *∗*%46	36 M/ 66 F	18-64, less than 110 lb weight	*∗*Intranasal ketorolac (31.5 mg ketorolac tromethamine) + nitrous oxide/oxygen (10 min before IANB and 20 min after intranasal ketorolac) *∗*Placebo (31.5 mg intranasal bacteriostatic 0.9% sodium chloride(saline) + nitrous oxide/oxygen (10 min before IANB and 20 min after intranasal saline)	Heft-parker vas; success=no pain or weak/mild pain ability to access and instrument	No significant difference	high

Sakhaeimanesh et al. 2017	Prospective Double-blind (++) Randomized (no explanation) Placebo-controlled	no	no	Heft-parker vas (96±30.2 and 101±30)	*∗*1.6 mL 4% articaine with 1:200000 Epinephrine + 0.2 mL ketamine hydrochloride (50 mg/mL) *∗*1.6 mL 4% articaine with 1:200000 Epinephrine + 0.2 mL normal saline (Placebo)	Failure-If the pain rating was moderate or greater during treatment	42 Mandibular posterior teeth (molar or premolar) *∗*%55 *∗*%42.9	24 M/ 18 F	19-56	*∗*1.6 mL 4% articaine with 1:200000 Epinephrine + 0.2 mL ketamine hydrochloride (50 mg/mL) *∗*1.6 mL 4% articaine with 1:200000 Epinephrine + 0.2 mL normal saline (Placebo)	success=Heft-parker vas no or mild on access cavity preparation and initial instrument placement	No significant difference	medium

Stanley et al. 2012	Prospective Double-blind (++) Randomized (6-digit random number) Placebo-controlled	yes	yes	Heft-parker vas, moderate or severe	*3.6 mL* of 2% lidocaine with 1:100,000 epinephrine	Topical anesthetic gel The patients who reported moderate or severe pain (VAS rating >54 mm) during access into dentin or when entering the pulp chamber -failure	100 Mandibular posterior teeth (molar or premolar) 39 *∗*%50 *∗*%28	43 M/ 57 F	Means 33±11 and 35±13 not younger than 18	*∗*inhalation regimen of nitrous oxide/oxygen mix 5 min before the IANB *∗*inhalation regimen of room air/ oxygen mix (placebo) 5 min before the IANB	success = ability to access and clean and shape the canals without pain (VAS score of 0) or mild pain (VAS rating ≤54 mm).	administration of 30%–50% nitrous oxide resulted in a statistically significant increase in the success	

Bigby et al. 2007	Prospective Single-blind (patient? +) Randomized (5-digit random number) Controlled ???	no	no	Heft-parker vas, (104±36 and 103±31)	*∗*1.8 ml of 36 mg of lidocaine with 18 *μ*g of epinephrine *∗*3.6 ml of 36 mg of lidocaine with 18 *μ*g of epinephrine plus 36 mg meperidine with 18 *μ*g of epinephrine	Topical anesthetic gel long buccal nerve injection using a quarter of a cartridge of 2% lidocaine with 1:100,000 epinephrine The patients who reported moderate or severe pain (VAS rating >54 mm) within dentin, entering the pulp chamber, or initial file placement-failure	50 Mandibular posterior teeth (molar or premolar) 8 *∗*%26 *∗*%12	20 M/ 28 F	20-53	*∗*1.8 ml of 36 mg of lidocaine with 18 *μ*g of epinephrine *∗*3.6 ml of 36 mg of lidocaine with 18 *μ*g of epinephrine plus 36 mg meperidine with 18 *μ*g of epinephrine	success = ability to access and instrument the tooth without pain (VAS score of 0) or mild pain (VAS rating ≤54 mm).	No significant difference	high

**HYPEROSMOLAR SOLUTIONS**													

Kreimer et al. 2012 Study 1 Mannitol: natural diuretic	Prospective Single-blind (patient?+) Randomized (4-digit computer random number) Controlled	yes	no	Heft-parker vas (89±43 and 92±53)	*∗*3.18 mL of lidocaine (63.6 mg) with 31.8 *μ*g Epinephrine *∗*3.18 mL of lidocaine (63.6 mg) with 31.8 *μ*g epinephrine plus 1.82 mL of 0.5 mol/L mannitol *(5 mL)*	If the patient felt pain, Heft–Parker VAS moderate or severe-failure	55 Mandibular posterior teeth (molar or premolar)	25 M/ 30 F	19-60	*∗*3.18 mL of lidocaine (63.6 mg) with 31.8 *μ*g Epinephrine *∗*3.18 mL of lidocaine (63.6 mg) with 31.8 *μ*g epinephrine plus 1.82 mL of 0.5 mol/L mannitol *(5 mL)*	Heft-parker vas; success=no pain or weak/mild pain ability to access and instrument	No significant difference	medium

Kreimer et al. 2012 Study 2 Mannitol: natural diuretic	Prospective Single-blind (patient?+) Randomized (4-digit computer random number) Controlled	yes	no	Heft-parker vas (73±36 and 71±41)	*∗*1.9 mL of lidocaine (76.4 mg) with 36 *μ*g Epinephrine *∗*1.9 mL of idocaine (76.4 mg) with 36 *μ*g epinephrine plus 1.1 mL of 0.5 mol/L mannitol *(3mL)*	If the patient felt pain, Heft–Parker VAS moderate or severe-failure	51 Mandibular posterior teeth (molar or premolar)	15 M/ 36 F	18-59	*∗*1.9 mL of lidocaine (76.4 mg) with 36 *μ*g Epinephrine *∗*1.9 mL of idocaine (76.4 mg) with 36 *μ*g epinephrine plus 1.1 mL of 0.5 mol/L mannitol *(3mL)*	Heft-parker vas; success=no pain or weak/mild pain ability to access and instrument	addition of 0.5 mol/L mannitol to 1.9 mL of lidocaine (76.4 mg) with epinephrine resulted in a statistically higher success rate	medium

**ANTIHYPERTENSIVE MEDICATIONS AND MAGNESIUM SULPHATE**													

Shadmehr et al. 2017 antihipertansif	Prospective Double-blind (++) Randomized (5-digit computer random number, sealed opaque envelope) Controlled	yes	Hemodynamic parameters were continuously monitored by an electrocardiogram monitor	Heft-parker vas (103±19 and 102±17)	*∗*1.8 mL of 2% lidocaine with clonidine (15 lg mL^−1^) *∗*1.8 mL of 2% lidocaine with epinephrine (12.5 lg mL-^1^) (Control group)	topical anesthetic gel At each step, when patients reported moderate to severe pain (>54 mm), the IANB was considered to have failed.	100 (98) 43 *∗*%59 *∗*%29	49 M/ 51 F	18-56	*∗*1.8 mL of 2% lidocaine with clonidine (15 lg mL^−1^) *∗*1.8 mL of 2% lidocaine with epinephrine (12.5 lg mL-^1^) (Control group)	Heft-parker vas; Success=absence of pain or only mild pain in all of the following steps (penetrate dentine, enter the pulp and advance instruments into the coronal part of the canal pulp)	clonidine group exhibited a significantly higher success rate	medium

Shetty et al. 2015	Prospective Double-blind (++) Randomized (6-digit random number) Placebo-controlled	no	no	Heft-parker vas (135.6±10.2 and 136.96±9.5)	1.8 mL 2% lidocaine with 1:100,000 epinephrine	moderate or severe pain (heft- parker VAS score, >54 mm) during access cavity preparation or initial file placement received supplemental anesthetic injection-failure	100 mandibular posterior tooth (premolar or molar) *∗*%58 *∗*%32	42 M/ 58 F	Means 33.48±3.8 and 31.8±4.4 not younger than 18	*∗*injection of 1 mL magnesium sulfate USP 50% -60 min before IABN *∗*Injection of 1 mL distilled water (placebo) -60 min before IABN	Heft-parker vas, Success= access cavity preparation or initial file Placement without pain (VAS score, 0 mm) or with mild pain (VAS score, ≤54 mm).	preoperative administration of 1 mL magnesium sulfate USP 50% resulted in statistically significant increase in success of IAN block compared with placebo.	high
